# ERP differences between processing of physical characteristics and personality attributes

**DOI:** 10.1186/1744-9081-8-49

**Published:** 2012-09-11

**Authors:** Fanchang Kong, Yan Zhang, Hong Chen

**Affiliations:** 1 Key Laboratory of Cognition and Personality (Ministry of Education) and School of Psychology, Southwest University, Chongqing, China; 2 Department of Educational Science, Mianyang Normal University, Sichuan, China

## Abstract

**Background:**

Limited data from behavioral and brain-imaging studies indicate that personality traits and physical characteristics are processed differently by the brain. Additionally, electrophysiological results of studies comparing the processing of positive and negative words have produced mixed results. It is therefore not clear how physical and personality attributes with emotional valence (i.e., positive and negative valence) are processed. Thus, this study aimed to examine the neural activity associated with words describing personality traits and physical characteristics with positive or negative emotional valence using Event Related Potentials (ERPs).

**Methods:**

A sample of 15 healthy adults (7 men, 8 women) participated in a computerized word categorization task. Participants were asked to categorize visual word stimuli as physical characteristics or personality traits, while ERPs were recorded synchronously.

**Results:**

Behavioral reaction times to negative physical stimuli were shorter compared to negative personality words, however reaction times did not significantly differ for positive stimuli. Electrophysiological results showed that personality stimuli elicited larger P2 and LPC (Late Positive Component) amplitudes compared to physical stimuli, regardless of negative or positive valence. Moreover, negative as compared with positive stimuli elicited larger P2 and LPC amplitudes.

**Conclusion:**

Personality and physical stimuli were processed differently regardless of positive or negative valence. These findings suggest that personality traits and physical characteristics are differentially classified and are associated with different motivational significance.

## Background

A large body of evidence suggests that physical characteristics exert a powerful influence on our first impressions of people [1-5]. The physical characteristics manipulated in previous studies typically included physical beauty and unattractiveness, or “ugliness.” Previous results indicate that physical beauty was perceived as a sign of inner, spiritual and moral beauty [6-8]. Several studies have demonstrated that physically attractive individuals are thought to possess more sociably desirable personalities and higher moral standards than those who are physically unattractive [3-5]. This phenomenon has been called the “beauty is good” stereotype and has been shown to have a moderate effect size in a meta-analysis conducted by Eagly and colleagues [9]. On the other hand, ugliness has been shown to have just as significant an impact [10], and similarly, perceptions of physical ugliness are thought to be associated with perceptions of inner ugliness. A recent study testing the “ugly is bad” stereotype, asked participants to evaluate women’s faces with words describing personality traits. This study demonstrated that unattractive faces were thought to possess more undesirable characteristics as compared with faces considered average or above-average in attractiveness [4]. As described above, individuals seem to infer personality attributes-both positive and negative-of other individuals based on their physical appearance.

In addition, brain-imaging findings support the reality of the “beauty is good” stereotype [5]. In one experiment, female subjects were asked to evaluate images of faces using positive and negative personality words during fMRI recording. Results demonstrated that the stereotype reflected an approach response toward beauty and goodness mediated by medial orbitofrontal cortex, a brain region that has been implicated in positive emotions and reward processing. Moreover, the stereotype also reflected an avoidance response away from unattractiveness and badness mediated by the insular cortex, a brain region commonly associated with negative emotions and pain. This suggests that there is brain activity common to the processing of both positive physical (e.g., beauty) and positive personality (e.g., good) attributes as well as negative physical (e.g., ugliness) and negative personality (e.g., bad) attributes.

Importantly, it seems that positive and negative personality traits and positive and negative physical attributes were processed differently by the brain. Specifically, pictures portraying ugliness have been associated with selective increased activation in the superior parietal gyrus compared with pictures portraying beauty. In contrast, pictures portraying beauty have been associated with selective increased activation of the right amygdala compared to pictures portraying ugliness [11]. In addition, both Western and Chinese individuals have similar tendencies towards beauty advocacy and ugliness suppression in personality traits, and negative personality traits induced individuals’ greater emotional reaction (e.g., flout, hate) compared to positive personality traits [12]. However, it is not clear how the physical and personality attributes that were embedded with emotional valence were processed in the temporal course.

Although no research has examined the neural basis of physical and personality attributes in temporal processing specifically, a great number of ERP studies have investigated the electrophysiological correlates of emotional stimuli processing. Most studies of emotional and neutral stimuli suggest greater brain response to emotional as compared to neutral stimuli [13]. Specially, some studies suggested both negative and positive emotional stimuli elicited larger EPN (Early Posterior Negativity) [14-18], P2 [19,20] LPC (Late Positive Component) [15,16,18] and/or LPP (Late Positive Potential) [21,22] amplitudes compared to neutral stimuli. Other studies suggested that compared to neural stimuli, only positive stimuli elicited larger EPN [18], LPC [18] or LPP [19,23] amplitudes, while another set of studies showed that only negative stimuli, elicited larger EPN [24], LPC [15,25-28] or LPP [24,29] amplitudes. These findings indicate that emotional (negative and/or positive) stimuli elicited larger amplitudes compared to neutral stimuli, although some discrepancies remain.

Some studies have directly compared the impact of positive versus negative stimuli, yielding further mixed results. In some cases, negative stimuli in contrast to positive stimuli elicited larger P2 [26,27], LPC [17,20,28], or LPP amplitudes [24,29], while in other cases positive stimuli in contrast to negative stimuli elicited larger EPN [17] and LPC amplitudes [15,17,18]. Still, other studies have found no difference in brain response to positive or negative stimuli, when both pictures [21,22] and words [15] are used as stimuli. Thus, the available findings are inconclusive, and the neural correlates of negative and positive stimuli processing warrant further investigation.

Event-related potentials (ERPs) are characterized by superior temporal resolution and can demonstrate the underlying neural correlates associated with processing different stimuli at different times. Available findings suggest that emotional stimuli enhance cortex responses at both early (such as P2, EPN) and late (such as LPC) ERP effects of word processing [13,20]. In regard to components, EPN is thought to reflect sensory encoding processes [14-18,23], and P2 indexes both rapid attention capture by emotional words [14,15,17] and rudimentary semantic stimulus classification [17]. The late positivity to emotional stimuli is modulated by their intrinsic motivational significance and evaluative context of stimuli presentation [21,22]. The size of the amplitude reflects underlying emotional reactivity and emotion regulatory function [30]. Moreover, emotional words and pictures are processed differently [15], although there are some common neural correlates (e.g., LPC). Words, but not pictures, help to successfully distinguish physical characteristics from personality traits [3-5]. In addition, a series of studies of the “beauty is good” stereotype have showed that negative words are processed differently from positive words, regardless of whether they describe physical or personality attributes, on both behavioral and brain-imaging measures [4,5]. However, the extent to which physical and personality attributes are processed differently and whether the process is modulated by the emotional value remains unclear. Therefore, the present study used ERPs to identify the neural basis of the evaluation of physical and personality words with emotional valence. Based on considerations above, we hypothesized that personality traits and physical characteristics would be processed differently in early and late ERP potentials regardless of negative or positive valence. Moreover, we hypothesized that negative and positive words would be processed differently in early and late ERP potentials.

## Methods

### Participants

Fifteen native Chinese students of Southwest University (seven males, mean age: 22.1 years and eight females, mean age: 21.1 years) participated in this study. Participants were questioned with regard to their physical, visual, and medical status, and handedness was determined. Each participant was healthy, right-handed and had normal vision. All participants provided signed informed consent. The study was approved by the Academic Committee of School of Psychology, Southwest University, China.

### Experimental stimuli

The stimulus words employed in our study were standardized as follows. First, 60 undergraduate students were asked to generate words used to describe physical and personality attributes, and these words were then combined with those used in previous studies [31-34]. In total, we collected 182 words, including 91 words describing physical characteristics and 91 words describing personality attributes. Second, a sample of 125 college students (60 male, 65 female) were recruited to rate the words on dimensions of arousal and familiarity using a 9-point Likert scale (1 = “extremely unexciting, extremely unfamiliar” to 9 = “extremely exciting, extremely familiar”), and participants also indicated whether each word represented a personality and/or physical attribute (1 = “yes;” 2 = no). Finally, the mean and standard deviation for dimensions of arousal and familiarity were calculated and the percentage of people who rated each word as a personality and/or physical attribute was determined.

The visual stimuli were 2-character Chinese words, including 60 words describing physical characteristics (e.g., slender, fat) and 60 words describing personality attributes (e.g., honest, evil), selected from the original bank of 182 words. For each subcategory, half of the words were associated with positive attributes while the other half were associated with negative attributes. The stimulus was presented in white Song style Chinese characters with 24 point font on a black background, resulting in a stimulus height of 2 cm and width of 3 cm on the screen. Both personality and physical words were matched for arousal, (5.44 ± 0.36 vs. 5.50 ± 0.49), familiarity (5.81 ± 0.82 vs. 5.82 ± 0.61), word frequency (.015 ± .01 vs. .017 ± .01) and lexical structure (18.28 ± 4.33 vs. 18.23 ± 4.99). Word frequency (1/1,000,000) was controlled using frequency counts for written language from Modern Chinese Language for Common Use [35]. They were also similar to one another in size, background, contrast grade, brightness, and other physical properties.

### Procedures

Prior to the experiment, participants were informed that the aim of the study was to test their ability to categorize words using a computer program. Participants were seated comfortably in an acoustically isolated room approximately 90 cm from the computer screen, with the horizontal and vertical angles below 6°. The screen resolution was 72 pixels per inch throughout the experiment. The computer task contained two blocks, each block consisting of 120 trials. The trials were presented randomly within each block, and rest intervals were built into the program to decrease the effect of fatigue. Stimulus words were presented in the center of a gray background. To increase the total number of trials, each word was presented twice during the experiment. Each trial was initiated by a 500 ms display of a white cross on the gray computer screen; the target word was then presented for 2000 ms until the response was made. The following trial appeared after a 500 ms black screen. The subject was asked to press “1” if the target word was a physical characteristic and press “2” if the target word was a personality attribute. Prior to experimental trials, a practice session including 8 trials was used to familiarize participants with the task.

### ERP recording and analysis

The EEG was recorded from 64 scalp sites using tin electrodes fitted in an elastic cap (Brain Products), with the linked reference on the left and right mastoids (averaged mastoid reference) [36], and a ground electrode placed on the medial frontal aspect. Eye movements were monitored with supra- and infra-orbitally electrodes and with electrodes on canthi. Impedance was maintained below 5 kΏ. EEG and electro-oculogram (EOG) were filtered from 0.01 to 100 Hz. A 30 Hz digital low pass filter was applied off-line to the continuous EEG data. After rejecting those trials with eye movements, blink, motion or other artifacts at each channel, the averaging of ERPs was computed off-line with computer algorithms. Trials with EOG artifacts with peak-to-peak deflection exceeding ± 80 μV and those contaminated with artifacts were excluded from averaging.

The resulting averages were based on correct responses in each separate condition [36]. The correct trials in each condition were as follows: Negative physical characteristics in 51 trials, positive physical characteristics in 45 trials, negative personality attributes in 54 trials, positive personality attributes in 52 trials. ERP waveforms were time locked to the stimuli onset, and the averaging epoch was 1100 ms, including a 200 ms pre-onset baseline. According to the grand average map, topographical maps and previous research [14], P2 was analyzed as averaged activity within the time window of 170–250 ms. The following electrodes were selected for statistical analyses of P2: F3, F4, Fz, FC3, FCz, FC4, C3, Cz, C4 [27]. Moreover, in the time window of 400–700 ms, LPC was analyzed in the following electrodes: CP3, CP4, CPz, Pz, P3, P4, POz, PO3, PO4, Oz [15,17,37,38]. Repeated measures ANOVAs were conducted on the amplitudes (baseline to peak) and peak latencies (from stimulus onset to the peak of the components) of P2. The ANOVA factors included attribute type (physical and personality), valence (positive and negative) and electrode site. The average amplitude of LPC was also measured. In the analysis of hemisphere effect, all the electrodes were grouped into left or right hemisphere except for middle electrodes (Fz, FCz, Cz, Pz and POz). Then, hemisphere variable was used instead of electrodes as a factor in three-factor repeated measures analysis. For the behavioral data, only correctly answered trials were analyzed in mean reaction times (RTs). For all analyses, *P*-values were calculated for deviations from sphericity. Significant main effects and interactions were followed by simple effects analyses and pairwise comparisons. The Greenhouse-Geisser method was applied to all repeated measures with greater than one degree of freedom [39]. In addition, the Bonferroni method was used for post-hoc pairwise comparisons.

## Results

### Behavioral data

**Figure 1:**
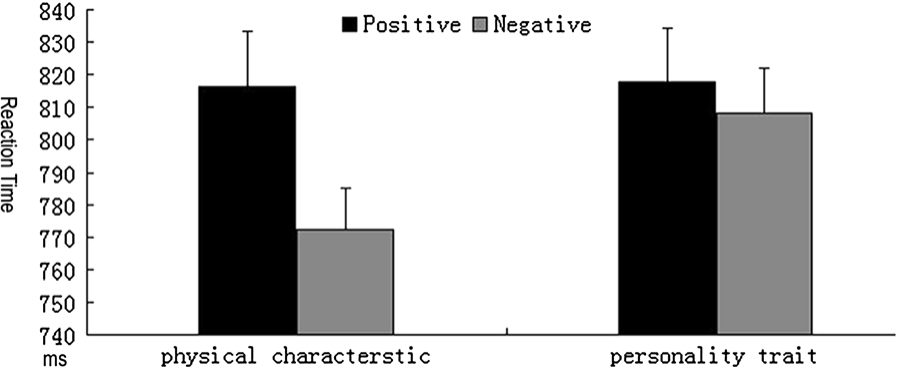
Reaction times of personality trait and physical characteristic words with positive and negative valences.

The repeated measures ANOVA was performed for RTs and revealed that the interaction of attribute type x valence was marginally significant [F (1, 14) =5.801, P = 0.05]. Simple analysis showed that the RTs for the negative personality words were significantly longer than those of negative physical words [F (1, 14) =2.451, P <0.05], but no significant difference emerged between positive physical and personality words [F (1, 14) =0.066, P >0.05] (See Figure 1). Additionally, the main effect of valence was significant [F (1, 14) =2.451, P <0.05]. The RTs for positive words (816.91 ± 14.21) were significantly longer than those of the negative words (790.16 ± 11.13). [F (1, 14) = 1.764, P >0.05]. For accuracy rate, results showed significant main effects of attribute type and valence [F (1, 14) = 18.556, P <0.01; F (1, 14) = 5.721, P <0.05], and no significant interaction of attribute type x valence [F (1, 14) = 3.898, P <0.05]. Post-hoc tests showed that subjects performed better for physical characteristics (0.88 ± 0.02%) than personality traits (0.81 ± 0.02%), and additionally better for negative stimuli (0.88 ± 0.02%) as compared with positive stimuli (0.82 ± 0.02%).

### Electrophysiological results

**Figure 2:**
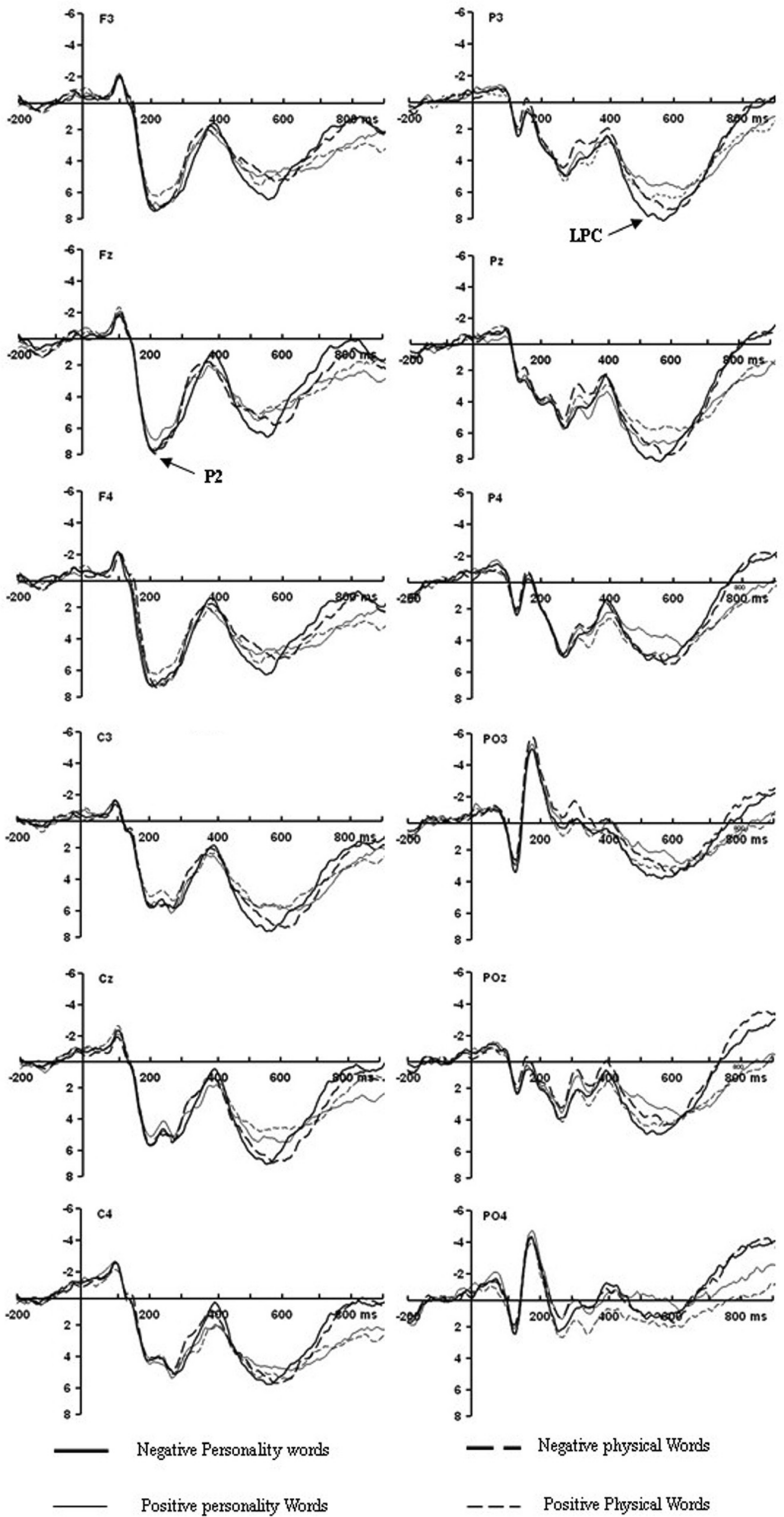
Grand average waveform of the stimulus-locked ERPs for P2 and LPC at F3, Fz, F4, C3, Cz, C4, P3, Pz, P4, PO3, POz, PO4 for negative personality (thick solid lines), positive personality (thin solid lines), negative physical (thick dotted lines) and positive physical (thin dotted lines) words.

**Figure 3:**
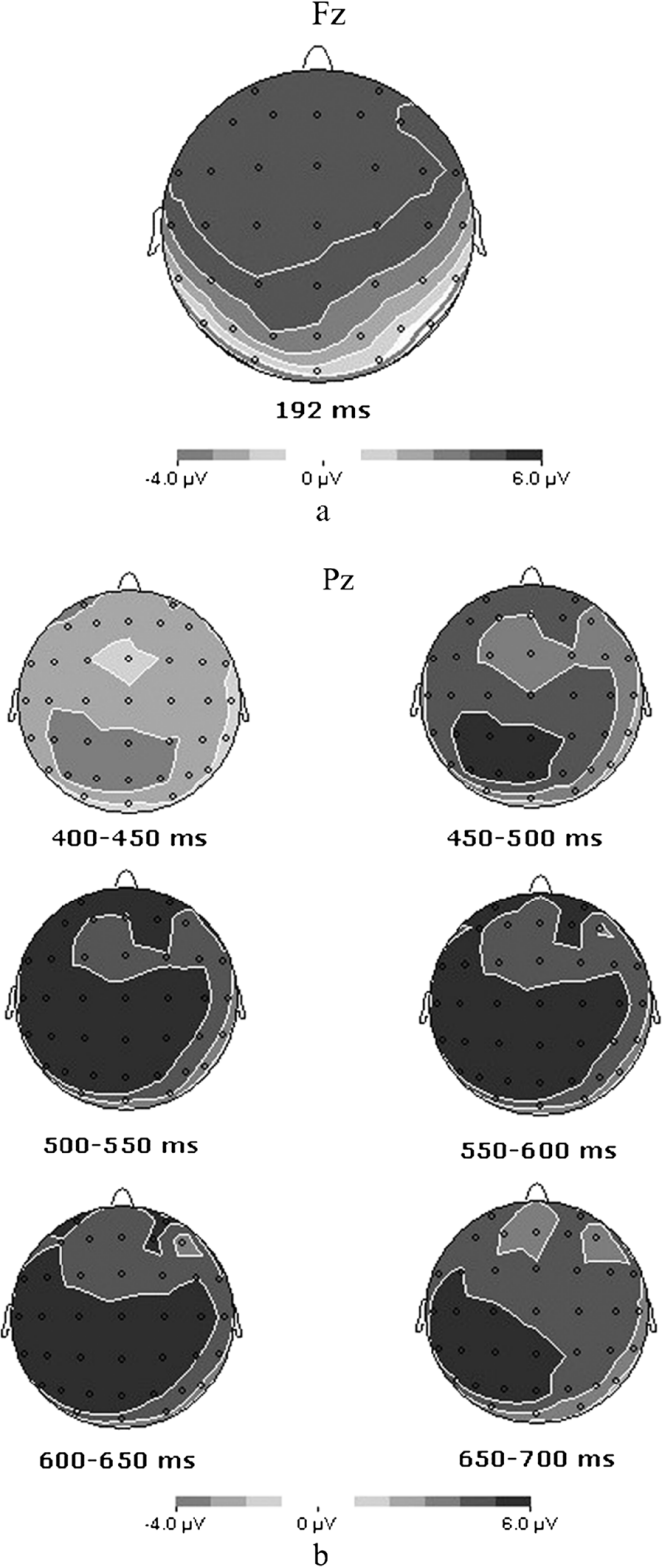
**Top view of voltage distribution maps showing the whole brain activity about stimulus type (personality versus physical). a**: the P2 voltage distribution at 192 ms at FCz. **b**: the LPC voltage distribution in the 400–700 ms at Pz.

As shown in the grand-average waveforms (See Figure 2), a repeated measures ANOVA was conducted on averaged amplitudes in the 170–250 ms interval. Results showed that the main effect of attribute type was significant [F (1, 14) = 5.636, P <0.05]. Post-hoc tests showed personality words (8.088 ± 0.994) elicited larger P2 amplitude as compared to physical words (7.158 ± 1.008). The main effect of valence was also significant [F (1, 14) = 15.536, P <0.01]. Negative words (7.984 ± 1.007) elicited larger P2 amplitude as compared to positive words (7.261 ± 0.965) (See Figure 3). Other effects were not significant. Moreover, no significant difference was recorded in latencies of P2 component in four conditions. The results of repeated measures ANOVA on hemisphere effects were not significant.

In the time window of 400–700 ms after stimuli onset, results of repeated measures ANOVA showed significant main effects of attribute type [F (1, 14) = 25.554, P <0.01], valence [F (1, 14) = 9.245, P <0.01] and electrode site [F (2.774, 38.833) = 3.688, P <0.05] (See Figure 2; Figure 3). Post-hoc tests showed that personality words (6.309 ± 0.640) elicited larger LPC amplitude compared to physical words (3.514 ± 0.833), and negative words (5.456 ± 0.674) elicited larger LPC compared to positive words (4.367 ± 0.748). Significant effects for attribute type x valence [F (1, 14) = 6.615, P <0.05], attribute type x electrode site [F (3.739, 52.352) = 3.522, P <0.05] and attribute type x valence x electrode site [F (3.796, 53.137) = 4.212, P <0.01] were also found. Simple analysis of attribute type x valence x electrode site effect showed that for negative words, personality trait words elicited larger LPC compared to physical characteristics at Center-Parietal electrodes. For positive words, personality trait words elicited larger LPC compared to physical characteristics at Parietal-Occipital electrodes (See Table 1; Figure 3). To examine the hemisphere effects, a repeated measures ANOVA was conducted. Results showed that main effect of hemisphere was significant [F (1, 14) = 8.820, P <0.01]. Post-hoc tests showed that LPC on the left hemisphere (5.882 ± .730) was larger as compared to LPC on the right hemisphere (4.432 ± .677).

**Table 1:** Results of simple analysis of attribute type × valence × electrode site effect on LPC amplitudes

**Electrode**	**MD(N)**	**SE(N)**	***p*(N)**	***F*(N)**	**MD(P)**	**SE(P)**	***p*(P)**	***F*(P)**
CP3	2.68	0.977	.016	2.740*	1.727	0.928	.084	1.861
CPz	1.39	0.689	.063	2.017	0.988	0.768	.219	1.286
CP4	1973	0.73	.017	2.703*	3.671	1.203	.009	3.052**
P3	2.167	0.541	.001	4.005**	2.162	1.316	.123	1.643
Pz	2.014	0.837	.031	2.406*	3.37	0.911	.002	3.699**
P4	2.473	0.887	.015	2.788*	2.565	1.332	.075	1.926
P03	2.425	0.902	.018	2.688*	5.168	0.601	0	8.599**
POz	2.118	0.995	.051	2.129	5.425	0.62	0	8.750**
P04	1.293	0.98	.208	1.319	6.201	0.566	0	10.956**
Oz	1.155	0.939	.239	1.230	4.935	0.531	0	9.294**

* means significant at the .05 level (2-tailed); ** means significant at .01 level (2-tailed).

Note: MD = Mean Difference (personality trait minus physical characteristic); SE = Std. Error. The left panel for negative personality and physical words and the right panel for positive personality and physical words.

## Discussion

The present study examined the neural correlates underlying the processing of personality traits and physical characteristics with emotional valence. Behavioral results showed that responses to negative physical words were faster as compared to negative personality words, but the same did not hold true for positive words. Electrophysiological results showed that personality words elicited larger P2 and LPC compared to physical words for both negative and positive words. Moreover, negative stimuli elicited larger P2 and LPC than did positive stimuli. These results were consistent with previous findings [13], cortical responses to emotional stimuli were apparent from early assembly of visual stimuli (around 200 ms) to attentional allocations (around 300 ms) and elaborated processing (more than 300 ms). Therefore, results from our study reflected that the response to personality attributes differed significantly from the response to physical attributes, from early attention allocation to later evaluation of the stimuli’s meaning.

Behavioral results of the present study showed that only responses to negative physical words were faster than those to negative personality words, which was partially consistent with previous findings [26]. For example, Yuan and his colleagues reported that responses to extremely negative faces were faster compared to moderately negative and neutral faces, however reaction times did not significantly differ for positive faces. One possible interpretation of these results is that people are more sensitive to negative stimuli compared to neutral and positive stimuli due to evolutionary adaption to threatening environments. In this study, words, rather than pictures, were used. Negative personality traits provided more threatening information [12] when compared to negative physical characteristics, but responses to negative physical characteristics were faster compared to negative personality traits. One possible interpretation of this discrepancy is that procedural differences, such as the stimulus category or experimental task used in previous study, led to the incongruous findings [26]. Future investigation should help clarify the root of these differences.

The electrophysiological data revealed that personality words elicited larger P2 amplitudes than did physical words in the time window of 170–250 ms. Numerous studies have suggested that P2 effect in early emotional stimuli processing consists of rapid attention capture by emotional words [14,15,17] and rudimentary semantic stimulus classification [17]. Results from this study suggested that more attention resources were recruited by personality words relative to physical words in category tasks. The likely interpretation was that personality and physical words were two separate classifications, and brain responses to both stimuli were also different in rapid processing. Another probable interpretation was that personality traits, regardless of positive or negative valence, induced a more intense emotional response compared to physical characteristics due to greater mental significance of personality traits. Moreover, negative words also elicited larger P2 amplitudes compared to positive words, regardless of whether they were in response to personality or physical attributes. In line with previous findings, negative words elicited larger P2 amplitudes than did positive words [27]. It was suggested that the difference in perceptual stages may derive from an intrinsic or learned bias of the perceptual system toward certain types of the stimuli [40]. In addition, considerable research has shown that people are more sensitive to negative stimuli in contrast to positive or neutral stimuli due to survival and adaption [26-28]. In this study, both negative personality and physical attributes as stimuli pose a threat to survival and adaptation compared to positive personality and physical attributes. Therefore, enhanced brain responses were shown toward negative stimuli as compared to positive stimuli.

As has been well established, emotional LPC is regarded as a component associated with more elaborate, task-dependent processing of emotional words [15]. Moreover, late positivity to emotional stimuli is modulated by their intrinsic motivational significance and the evaluative context of stimuli presentation [21,22]. In this case, amplitude size is thought to reflect underlying emotional reactivity and emotion regulatory function [30]. In this stage, more factors will be considered and more experiences will be referenced. Results from this study showed that personality traits elicited larger LPC compared to physical words with both negative and positive valence in the time window of 400–700 ms after stimuli onset. This indicated that both positive and negative personality words were processed more elaborate compared to physical words, perhaps suggesting that personality traits related to greater motivational significance and more cognitive resources in mean evaluation.

Interestingly, negative personality trait words elicited larger LPC compared to physical characteristic words at central-parietal electrodes (e.g., CP3, CP4), while positive personality trait words elicited larger LPC relative to physical characteristic words at parietal-occipital electrodes (e.g., PO3, PO4). This likely indicates that responses to positive and negative personality and physical words reflect, at least, the involvement of partially different neural correlates. Previous research found that individuals express an approach response toward beauty and goodness mediated by medial orbitofrontal cortex and an avoidance response away from unattractiveness and badness mediated by the insular cortex [5]. Thus, it was inferred that brain responses to positive and negative physical and personality words likely reflect separate cognitive process. For positive stimuli, amplitudes elicited by personality traits were larger than those elicited by physical characteristics due to larger reward significance. In contrast, for negative stimuli, amplitudes elicited by personality, in comparison to physical traits, were larger due to larger threatening significance. Furthermore, it was difficult to localize the specific differences of brain activation in processing personality and physical attributes with positive versus negative valence because ERPs have limited spatial resolution; however, significant differences in brain activation were detected. Future research should further investigate the specificity of these differences.

Consistent with previous research [17,20,28,41], negative words elicited larger LPC in comparison to positive words. Specially, negative personality and physical words elicited larger LPC compared to positive personality and physical words. As mentioned above, LPC was related to evaluation of meaning, and more elaborate processes. Thus, the results indicate that negative words were processed more elaborately than positive words. One explanation is that negative stimuli were interpreted as threatening, leading individuals to be more sensitive to negative stimuli than to positive stimuli, which is in line with a survival or evolutionary standpoint [26-28]. Finally, results of hemisphere effect analysis showed that LPC, but not P2, was larger on the left hemisphere compared to the right hemisphere in processing personality and physical attributes. Consistent with previous research [20], LPC was larger for words that presented in the left hemisphere relative to the right hemisphere, since words were processed in the left brain [42].

## Conclusion

This study is among the first to identify specific neural correlates involved in the evaluation of physical and personality words using ERPs. Results suggested that responses to negative physical words were faster compared to negative personality words, but this does not hold true for positive words. When compared to physical words, personality words elicited larger P2 and LPC amplitudes regardless of negative or positive valence. Moreover, negative personality and physical words elicited larger P2 and LPC amplitudes in comparison to positive personality and physical words. These results demonstrated that personality attributes differed significantly from physical characteristics, from the early attention allocation to later evaluation of the stimuli’s meaning. This helps us to further understand the inner relation of physical and personality attributes, and provides suggestions of judgments of incongruities of positive and negative words, such as a good person with an ugly appearance or bad person with a beautiful face. Future research should further examine the specific brain activations involved in processing personality and physical attributes with emotional valence. Additionally, further studies should help to understand why individuals show different responses to personality and physical words with a different valence.

## Competing interests

None of the authors have any competing financial interests.

## Authors’ contributions

FK participated in the conception, design and writing of the study. YZ prepared the data for analysis and advised on interpretation of the results. FK and YZ contribute equally in this study HC contributed to research design and modifications in subsequent drafts. All authors read and approved the final manuscript.
